# Supplementary material for: Prostate-specific antigen dynamics after neoadjuvant androgen-deprivation therapy and carbon ion radiotherapy for prostate cancer
Source: PLoS One. 2020 Nov 6;15(11):e0241636. doi: 10.1371/journal.pone.0241636 (PMC7647067; doi:10.1371/journal.pone.0241636)
Supplement: Attachment — Submitted filename: Responce to reviwers.docx [file pone.0241636.s002.docx]

Reviewer #1: The revised version is well re-written replying the comments of the reviewers. I feel that the revised manuscript is suitable for publication.

We thank the reviewer for evaluating our manuscript and for their encouraging comments.

Reviewer #2: Given the fact that the clinical significance of PSA bounce is unclear and its definition is various, I think such a prospective observational study is valuable. This manuscript is very important as the first report of PSA dynamics after CIRT with neoadjuvant ADT. I think this paper is worthy for publication.

We appreciate the reviewer’s feedback and are elated to hear this news.

Reviewer #3: Thank the authors for revising the manuscript; however, some problems remain in the revised manuscript.

We thank the reviewer for evaluating our manuscript. According to the suggestions, we have revised the manuscript as follows.

1. The topic of the manuscript is “Prostate-Specific Antigen Dynamics After Carbon Ion Radiotherapy for Prostate Cancer.” If the authors were not able to confirm whether the PSA dynamics was due to the effect of CIRT, ADT or both, the issue of this investigation should be “Prostate-Specific Antigen Dynamics After Neoadjuvant ADT and CIRT for Prostate Cancer.” Precisely, the study demonstrated the prostate-specific antigen dynamics after neoadjuvant androgen-deprivation therapy combined with carbon ion radiotherapy for intermediate risk prostate cancer patients.

We have revised the title to “Prostate-Specific Antigen Dynamics After Neoadjuvant Androgen-Deprivation Therapy and Carbon Ion Radiotherapy for Prostate Cancer,” as per their insight (page 1, lines 2–3).

2. Table 1 demonstrated that the duration of ADT were enormously diverse with median of 6.2 months (ranging from 2.3 to 116.9 months). Essentially, this arises the concern of its potential effects on the time period for PSA bounce or PSA failure. This issue stayed unexplained in the revised manuscript.

Tables 2 and 3 demonstrate that the ADT duration was not associated with the occurrence of PSA bounce and failure. This has been delineated in the Results section as follows:

ADT duration was not correlated with the occurrence of PSA bounce (p = 0.731) (page 16, lines 219–220).

ADT duration was not correlated with the occurrence of PSA failure (p = 0.614) (page 20, lines 258–259).

3. Only eight patients developed biochemical failure during follow-up of this cohort. The small number could reduce the power of statistical analysis, which should be estimated in the manuscript.

We agree with the reviewer’s comment that this small number of patients has contributed to a reduction of statistical power. We have acknowledged this as a limitation of our study, which is as follows:

In particular, the small number of PSA failure cases could reduce the power of the statistical analysis (page 26, lines 341–342).

4. Importantly, the statistical analyses have some fundamental problems even after revision. The results of the univariate analysis and Cox regression model of multivariate analysis provided in Table 2 and Table 3 were insufficient to demonstrate how those parameters were correlated with the time period for PSA bounce or PSA failure to happen. For example, if younger age and lower T stage were not clearly defined in the investigation, how did authors conclude that younger age and lower T stage were associated with the time period for PSA bounce or PSA failure to occur after CIRT?

Because CIRT requires advanced techniques and the patient number is limited compared with those who receive photon radiotherapy, this requires careful statistical analysis to investigate PSA dynamics to confirm its clinical impact.

In the present study, we solely aimed to determine the occurrence of PSA bounce, PSA failure, and related clinical factors; elucidation of the time period for PSA bounce or failure to occur was beyond the scope of this study. This has been stated in the manuscript as follows:

In this study, we identified the significant predictors of the occurrence of PSA bounce and failure. (page 4, lines 55–56).

The occurrence of PSA bounce and failure was correlated with younger age. (page 22, lines 273–274).
