# Supplementary material for: Prostate-specific antigen dynamics after neoadjuvant androgen-deprivation therapy and carbon ion radiotherapy for prostate cancer
Source: PLoS One. 2020 Nov 6;15(11):e0241636. doi: 10.1371/journal.pone.0241636 (PMC7647067; doi:10.1371/journal.pone.0241636)
Supplement: Attachment — Submitted filename: Responce to reviwer.docx [file pone.0241636.s003.docx]

Reviewer #4:

We thank the reviewer for evaluating our manuscript. According to the suggestions, we have revised the manuscript as follows.

“In the statistical analysis section, authors should give details on how the ROC curves were used to generate the optimal cutoffs (was it from univariable model? Or multivariable model?) or list any appropriate reference articles.”

We used Youden index to determine the optimal cut-off value. We have revised the statistical analysis section to clarify how the cut off value was generated (Page 11, lines 172-174) and a new reference have been added (ref number 29)

“Non-parametric receiver operating characteristic (ROC) curves were generated and Youden index (J = max [sensitivity + specificity – 1]) was used to determine the optimal cut-off values [29].”

“29) Conroy AL, Liles WC, Molyneux ME, Rogerson SJ, Kain KC. Performance characteristics of combinations of host biomarkers to identify women with occult placental malaria: a case-control study from Malawi. PLoS One. 2011;6(12)”
